# SnO_2_ Nanoparticles for Sensing and Bone Regeneration Application: Wet-Chemical and Plant-Based Green Synthesis, Spectroscopic Characterization, Photocatalytic, and SERS Activities

**DOI:** 10.3390/nano15241839

**Published:** 2025-12-05

**Authors:** Edyta Proniewicz, Olga Surma, Marta Gajewska, Marcin Molenda

**Affiliations:** 1 Faculty of Foundry Engineering, AGH University of Science and Technology, 30-059 Krakow, Poland; 2 Faculty of Chemistry, Jagiellonian University, Gronostajowa 2, 30-387 Krakow, Poland; olga.surma@doctoral.uj.edu.pl (O.S.); marcin.molenda@uj.edu.pl (M.M.); 3 Academic Centre for Materials and Nanotechnology, AGH University of Science and Technology, 30-055 Krakow, Poland

**Keywords:** tin dioxide nanoparticles (SnO_2_NPs), green synthesis, photocatalytic activity, surface-enhanced Raman scattering (SERS), L-phenylalanine adsorption

## Abstract

This study presents the synthesis and comprehensive characterization of tin dioxide nanoparticles (SnO_2_NPs). SnO_2_NPs were obtained using a conventional wet-chemistry route and an environmentally friendly green-chemistry approach employing plant extracts from rooibos leaves (*Aspalathus linearis*), pomegranate seeds (*Punica granatum*), and kiwifruit peels (family *Actinidiaceae*). The thermal stability and decomposition profiles were analyzed by thermogravimetric analysis (TGA), while their structural and physicochemical properties were investigated using X-ray diffraction (XRD), energy-dispersive X-ray spectroscopy (EDS), ultraviolet–visible (UV–Vis) spectroscopy, dynamic light scattering (DLS), Raman spectroscopy, and attenuated total reflectance Fourier-transform infrared (ATR-FTIR) spectroscopy. Transmission electron microscopy (TEM) confirmed the nanoscale morphology and uniformity of the obtained particles. The photocatalytic activity of SnO_2_NPs was evaluated via the degradation of methyl orange (MeO) under UV irradiation, revealing that nanoparticles synthesized using rooibos extract exhibited the highest efficiency (68% degradation within 180 min). Furthermore, surface-enhanced Raman scattering (SERS) spectroscopy was employed to study the adsorption behavior of L-phenylalanine (L-Phe) on the SnO_2_NP surface. To the best of our knowledge, this is the first report demonstrating the use of pure SnO_2_ nanoparticles as SERS substrates for biologically active, low-symmetry molecules. The calculated enhancement factor (EF) reached up to two orders of magnitude (10^2^), comparable to other transition metal-based nanostructures. These findings highlight the potential of SnO_2_NPs as multifunctional materials for biomedical and sensing applications, bridging nanotechnology and regenerative medicine.

## 1. Introduction

In recent decades, tin dioxide nanoparticles (SnO_2_NPs) have attracted considerable attention due to their unique physical properties, including high chemical stability and sensitivity, favorable mechanical properties, optical characteristics, and atmosphere-dependent electrical conductivity (under both oxidizing and reducing conditions) [1,2,3,4]. Their large surface-to-volume ratio enhances their chemical and biological activity, making them promising candidates for use as radical scavengers [5,6,7]. Furthermore, they are inexpensive and readily available. Consequently, SnO_2_NPs are widely used in gas sensors [8], supercapacitors [9], solar cells [10], rechargeable lithium-ion batteries [11] and other functional materials. More recently, their potential biomedical applications have been recognized. SnO_2_NPs exhibit notable antioxidants and antibacterial activities. For instance, they have been shown to inhibit the growth of both Gram-positive bacteria (e.g., *Streptococcus pyogenes*, *Staphylococcus aureus*, *Enterococcus faecalis*, *Bacillus subtilis*, and *Staphylococcus epidermidis*) and Gram-negative bacteria (e.g., *Klebsiella pneumoniae* and *Escherichia coli*) [12,13,14]. This antibacterial activity is attributed to their ability to penetrate bacterial membranes and inhibit cell proliferation, either by disrupting the cell wall via the production of reactive oxygen species (ROS) during the decomposition or through electrostatic interactions with the cell membrane [15,16]. In addition, several studies have demonstrated the potential of SnO_2_NPs in bone regeneration and repair [16,17,18,19]. The increasing clinical use of biomaterials for bone tissue engineering raises concerns regarding osseointegration and postoperative bacterial infections. Commonly used polymers, such as poly(L-lactic acid) (PLLA) and poly(methyl methacrylate) (PMMA), as well as ceramics (hydroxyapatite, HA) and metals (e.g., titanium), may be colonized by bacteria and are generally bioinert. In this context, SnO_2_NPs have been shown to modulate cellular behavior through electronic and chemical signaling [17,20,21,22]. Furthermore, SnO_2_NPs have demonstrated therapeutic potential against various cancer cell lines, including ovarian cancer, MCF-7 human breast cancer cells, U-2 OS human osteosarcoma cells, leukemia K562 cells, and cervical carcinoma cells [23,24,25,26,27,28].

Bulk SnO_2_ is a transparent n-type semiconductor (approximately 97% transparent across the visible spectrum) with a band gap of 3.6 eV (344 nm) at 300 K [29]. Upon heating, SnO_2_ undergoes oxidation, resulting in the formation of tin cations with unsaturated coordination (four instead of six). This transformation leads to the formation of a non-stoichiometric oxide. Thermal excitation of this oxide can produce paramagnetic species, such as V_O_^+^ (F-center) and Sn^3+^ [30]. In the absence of degeneracy, enhanced absorption at longer wavelengths is observed, allowing for the detection of surface species [31]. However, near-infrared (NIR) spectroscopy encounters challenges associated with free carriers (intraband transitions) and electron/hole donor transitions involving localized states. The concentration of these species depends on atmospheric conditions and temperature [30].

Various physical and chemical methods have been developed for the synthesis of SnO_2_NPs, including rapid tin oxidation [32], etching [33], hydrothermal synthesis, thermal decomposition [34], chemical vapor deposition [35], and sol–gel processing [36]. Many of these methods require surfactants or combustion aids, which may pose health risks and contribute to environmental contamination. Additionally, these methods often involve complex procedures or costly instrumentation. Therefore, there is a growing need to develop simple, cost-effective, and environmentally friendly synthesis routes capable of producing highly crystalline nanostructures with tunable properties and narrow particle size distributions. Plant-mediated green synthesis has emerged as a sustainable alternative that meets these requirements. Furthermore, such approaches align with the European Landfill Directive (1999/31/EC) and the Waste Framework Directive (2008/98/EC), which oblige EU member states to reduce biodegradable waste destined for landfills, including peels and seeds [37,38]. Industrial reuse of this waste can enable the production of natural additives, such as metal oxides, through environmentally benign routes.

**Figure 1:**
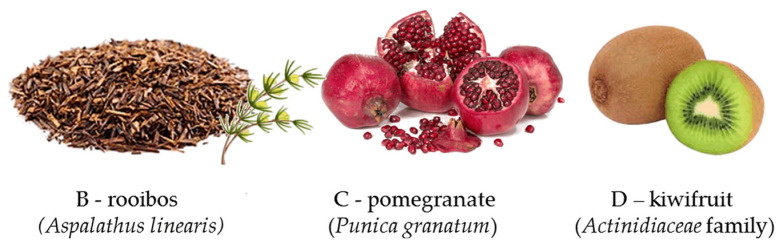
Plants readily available and used as reducing agents for the green synthesis of SnO_2_ nanoparticles (SnO_2_NPs).

In this study, SnO_2_ NPs were synthesized using both conventional wet chemical and green chemistry methods employing plant extracts derived from three sources: rooibos (*Aspalathus linearis*) leaves, pomegranate (*Punica granatum*) seeds, and kiwifruit (*Actinidiaceae* family) peels (Figure 1). These plants contain phytochemicals such as phenolic compounds, flavonoids, ellagitannins, and proanthocyanidins, which act as reducing and stabilizing agents during nanoparticle formation. The synthesized nanoparticles were characterized using various spectroscopic and microscopic techniques, including X-ray diffraction (XRD), Raman spectroscopy, attenuated total reflectance Fourier-transform infrared spectroscopy (ATR-FTIR), UV–Vis spectroscopy, and transmission electron microscopy coupled with energy-dispersive X-ray spectroscopy (TEM-EDS).

The photocatalytic performance of the SnO_2_NPs was assessed through the degradation of methyl orange (MO) at room temperature, and their recyclability was evaluated over multiple cycles. Additionally, surface-enhanced Raman scattering (SERS) spectroscopy was employed to investigate adsorption phenomena on the nanoparticle surfaces. The results were compared to identify the most efficient synthesis method for SnO_2_NPs, intended for use as additives in 3D-printed bone scaffolds.

## 2. Materials and Methods

### 2.1. Materials

Plant extracts were obtained from rooibos (*Aspalathus linearis*) leaves, pomegranate (*Punica granatum*) seeds, and kiwifruit (*Actinidiaceae* family) peels (Figure 1). Tin(II) chloride dihydrate (SnCl_2_·2H_2_O), tin(IV) chloride pentahydrate (SnCl_4_·5H_2_O), concentrated hydrochloric acid (HCl), ammonia solution (NH_4_OH), methyl orange (MeO), and L-phenylalanine (L-Phe) were purchased from Merck (Poznań, Poland). All reagents were of analytical grade and used without further purification.

### 2.2. Synthesis SnO_2_ Nanoparticles

**Wet chemical synthesis**

An ultrasonic cleaner was used to dissolve 25 g of SnCl_2_·2H_2_O in 20 mL of concentrated HCl and 10 mL of distilled water [39]. Ammonia solution (1:1, *v*/*v*) was then added until the pH reached 1.0, and the mixture was stirred for 10 min. Subsequently, a 2 M ammonia solution was added to adjust the pH to 2.5. The resulting precipitate was filtered, washed with distilled water, and dried at 120 °C for 12 h, followed by an additional 1 h at 180 °C.

**Green synthesis**

A green chemistry approach was employed using extracts from rooibos leaves, pomegranate seeds, and kiwifruit peels.

Kiwifruit extract: Peels were mixed with distilled water at a ratio of 1:10 (*w*/*v*) and heated to 60 °C for 60 min. The solution was then cooled and filtered. A 5 mL portion of the extract was added to 10 mM aqueous SnCl_4_ solution and stirred for 1.5 h at 100 °C. The obtained precipitate was filtered, washed with distilled water, and air-dried.

Pomegranate seed extract: 1 mL of fresh juice from crushed seeds was diluted to 50 mL with deionized water and filtered. Five milliliters of the filtrate were added to 20 mL of a 0.1 M aqueous SnCl_4_ solution. The mixture was stirred for 5 min at room temperature. The resulting precipitate was centrifuged, washed with distilled water, and dried at room temperature.

Rooibos extract: 8 g of powdered rooibos leaves were added to 450 mL of distilled water and left to stand for 48 h. The mixture was then filtered, and 4 g of SnCl_4_·5H_2_O was dissolved in 400 mL of the filtrate. After 10 min, the precipitate was filtered, washed with distilled water, and air-dried.

### 2.3. Spectroscopic Characterization of SnO_2_ Nanoparticles

**Thermogravimetric analysis (TGA)**

TGA was performed using a Mettler Toledo TGA/SDTA 851e instrument (Mettler-Toledo International Inc., Greifensee, Switzerland). Measurements were carried out under an air flow of 80 cm^3^ min^−1^ within a temperature range of 25–1000 °C and at a heating rate of 10 °C min^−1^.

**Calcination**

Calcination was conducted in a muffle furnace under controlled airflow. Based on TGA results, SnO_2_NPs synthesized from rooibos, pomegranate, and kiwifruit extracts were calcined at 700 °C, 600 °C, and 500 °C, respectively. Samples were heated at a rate of 5 °C min^−1^, maintained at the target temperature for 6 h, and then cooled to room temperature.

**Transmission electron microscopy (TEM) and EDS**

TEM images and energy-dispersive X-ray spectroscopy (EDS) data were obtained using a Tecnai G2 transmission electron microscope (FEI Company, Hillsboro, OR, USA) operating at 200 kV. The microscope was equipped with an EDX microanalyzer and a high-angle annular dark-field (HAADF) detector. Observations were carried out in bright-field (BF), selected-area electron diffraction (SAED), high-resolution TEM (HRTEM), and scanning transmission electron microscopy (STEM) modes.

**Dynamic light scattering (DLS)**

Particle size distribution was determined using a Zetasizer Nano ZS analyzer (Malvern Instruments Ltd., Malvern, Worcestershire, UK). The analyzer was equipped with an avalanche photodiode detector with >50% quantum efficiency at 633 nm and operated at 25 °C. A 0.1 wt% suspension of SnO_2_ NPs in distilled water was sonicated for 15 min at 20 W (continuous mode) using a Branson SFX250 ultrasonic homogenizer before measurement.

**UV–Vis spectroscopy**

UV–Vis absorption spectra were recorded using a Lambda 25 spectrophotometer (PerkinElmer, Inc., Shelton, CT, USA).

**X-ray diffraction (XRD)**

Powder XRD patterns were obtained using an Aeris diffractometer (PANalytical B.V., Almelo, The Netherlands) with Cu Kα_1_ radiation (λ = 1.5406 Å). Measurements were performed over an angular range of 10–80° (2θ).

**Raman spectroscopy**

Raman spectra were collected using an InVia Raman spectrometer (Renishaw, Wotton-under-Edge, Gloucestershire, UK) equipped with a Leica microscope (50× objective) and an air-cooled CCD detector. A diode laser emitting at 785 nm (10 mW output power) was used as the excitation source. Four accumulations were collected for each spectrum with a spectral resolution of 4 cm^−1^.

**ATR-FTIR spectroscopy**

ATR-FTIR spectra were recorded using a Thermo Scientific Nicolet 6700 spectrometer (Thermo Fisher Scientific, Waltham, MA, USA) equipped with a diamond ATR accessory. Measurements were performed at 4 cm^−1^ resolution with 500 scans per spectrum.

### 2.4. Photocatalytic Activity of SnO_2_ Nanoparticles

Photocatalytic activity was evaluated in a quartz cell containing an aqueous mixture of SnO_2_NPs, MeO, and distilled water in a molar ratio of approximately 1:4.5:8. The suspension (1 mL) was kept in the dark for 30 min to reach adsorption–desorption equilibrium before irradiation. Then, suspension was irradiated with 365 nm UV light (UV lamp model LP2536UV, Kamush UV Technology Co., Ltd., Guangdong, China, intensity 0.8 mW/cm^2^) for up to 180 min in 30 min intervals. Absorbance changes were monitored using a Lambda 25 UV–Vis spectrophotometer.

### 2.5. Surface-Enhanced Raman Scattering (SERS) Measurements

L-phenylalanine (L-Phe) was dissolved in deionized water (conductivity = 0.08 μS cm^−1^) to prepare a 10^−3^ mol L^−1^ solution. The SnO_2_NP suspension was mixed with the L-Phe solution and incubated for 24 h prior to measurement.

SERS spectra (spectral resolution = 4 cm^−1^) were recorded using an InVia Raman spectrometer (Renishaw) equipped with a Leica microscope (50× objective) and an air-cooled CCD detector. Excitation was provided by a 785 nm diode laser (10 mW output power, 40 s exposure per accumulation). Spectra were collected at three different locations on three separate drops of the SnO_2_ NP/L-Phe mixture (nine spectra in total). No spectral changes indicative of sample degradation were observed.

### 2.6. Spectral Analysis

Spectral data were analyzed using SpectraGryph software (version 1.2.15, Dr. Friedrich Menges, Oberstdorf, Germany, 2016–2020).

## 3. Results and Discussion

### 3.1. Spectroscopic Characterization

**Figure 2:**
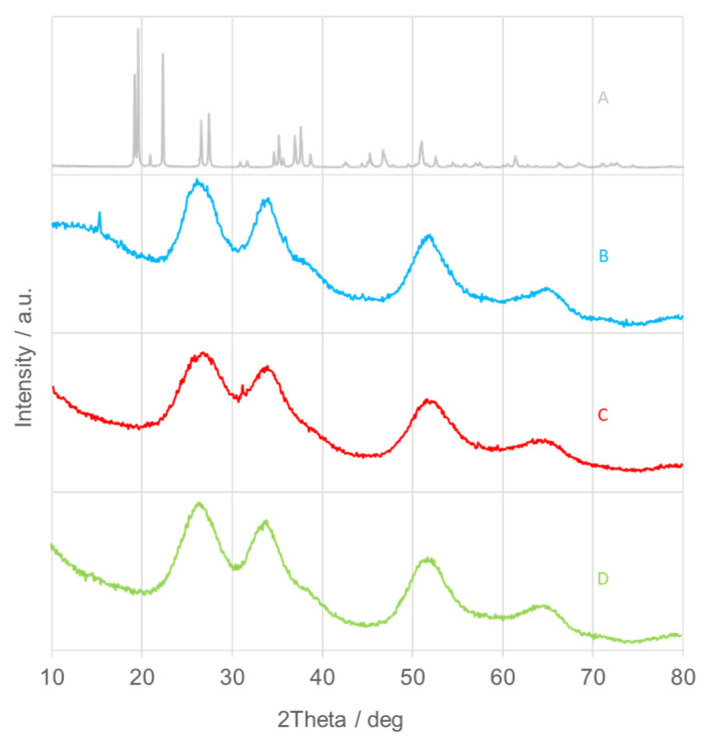
X-ray diffraction (XRD) patterns of as-prepared SnO_2_ nanoparticles (SnO_2_NPs) synthesized via (A) the wet-chemistry method and the green synthesis method using extracts from (B) rooibos leaves, (C) pomegranate seeds, and (D) kiwifruit peels.

Crystalline materials, characterized by a regular and periodic atomic arrangement, exhibit sharp and well-defined diffraction peaks in X-ray diffraction (XRD) patterns. In contrast, amorphous or poorly crystalline substances display broad and diffuse halos instead of sharp reflections. In this study, the XRD patterns of the as-synthesized SnO_2_NPs (Figure 2, traces B–D) indicate a low degree of crystallinity or an amorphous nature. The reduced crystallinity of the samples synthesized using plant extracts may result from local temperature increases during the exothermic hydrolysis of tin salts under alkaline conditions [40]. Trace A in Figure 2 corresponds to tin(II) hydroxychloride (Sn(OH)Cl) [39]. Accordingly, all samples were subjected to calcination for further structural investigation.

**Figure 3:**
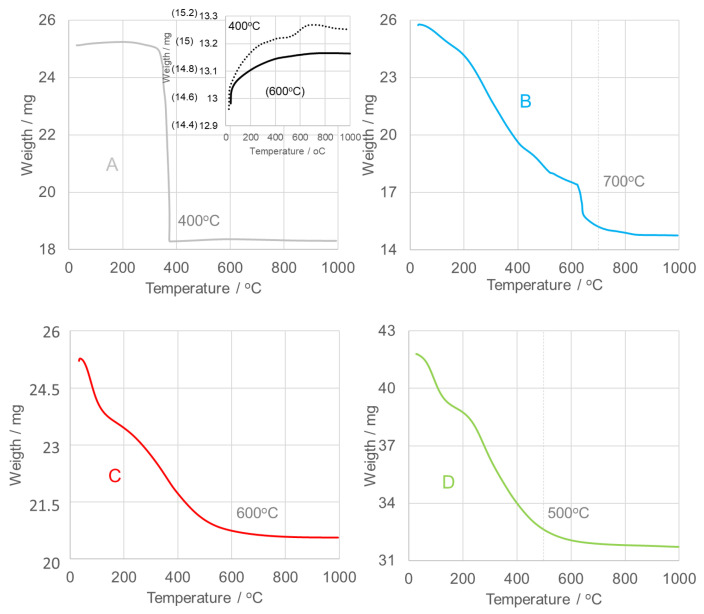
Thermogravimetric analysis (TGA) of as-prepared SnO_2_ nanoparticles (SnO_2_NPs) synthesized via (**A**) the wet-chemistry method and the green synthesis method using extracts from (**B**) rooibos leaves, (**C**) pomegranate seeds, and (**D**) kiwifruit peels.

Thermogravimetric analysis (TGA) was performed to determine the optimal calcination temperature. Figure 3 shows the TGA curves for all examined oxides. The SnO_2_NPs-A sample, synthesized via the wet chemical route, exhibits a distinct thermal decomposition profile compared with samples B–D, obtained through green synthesis using extracts from rooibos leaves (B), pomegranate seeds (C), and kiwifruit peels (D). Samples B–D show gradual three-step weight losses, while sample A undergoes a rapid mass reduction between 350–375 °C. An 18% weight loss in this region corresponds to the volatilization of tin as Sn^2+^ (Sn(OH)Cl) and formation of SnO_2_ [41]. A slight 1% increase in mass between 400–600 °C is attributed to oxidation processes, followed by a further 2% weight loss above 600 °C. Consequently, SnO_2_NPs-A were calcined at 400 °C (sample A’) and 600 °C (sample A).

**Figure 4:**
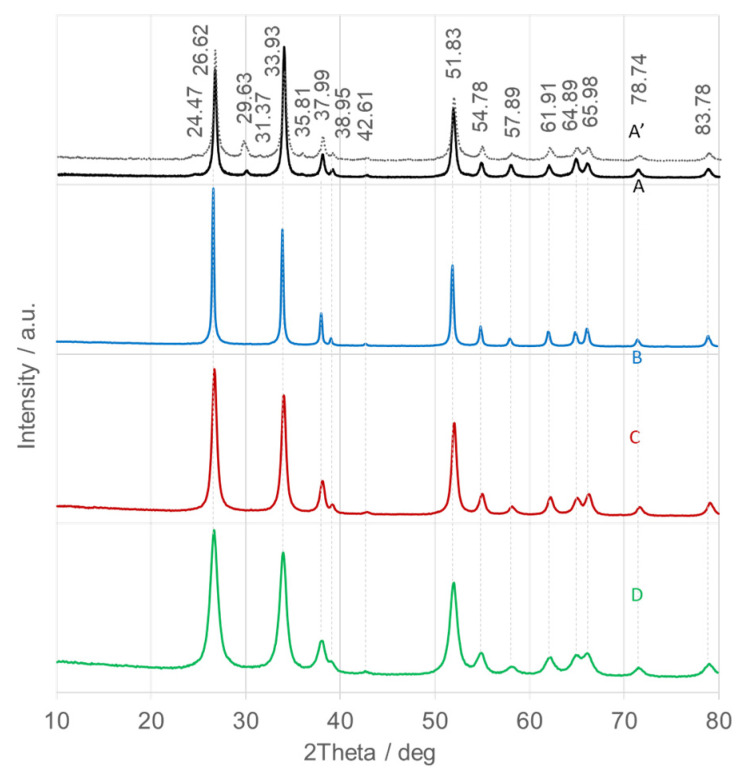
X-ray diffraction (XRD) patterns of calcined SnO_2_ nanoparticles (SnO_2_NPs) synthesized via the wet-chemistry method (A, calcined at 400 °C and A’, at 600 °C), and via the green chemistry method using extracts from (B) rooibos leaves, (C) pomegranate seeds, and (D) kiwifruit peels.

For samples B–D, stage I (~150 °C) is associated with dehydration, resulting in minor mass losses of 4% (SnO_2_NPs-B), 6% (SnO_2_NPs-C), and 6% (SnO_2_NPs-D). Stage II corresponds to gradual dehydroxylation occurring between 150–700 °C (SnO_2_NPs-B), 150–600 °C (SnO_2_NPs-C), and 150–500 °C (SnO_2_NPs-D), accompanied by oxidation of organic residues from the plant extracts, producing CO_2_ and H_2_O. The associated weight losses are 37%, 12%, and 16%, respectively. Stage III, beginning between 500–700 °C, involves less than 2% weight loss, indicating stabilization of the crystalline oxide phases. Based on these results, the calcination temperatures were set at 700 °C for SnO_2_NPs-B, 600 °C for SnO_2_NPs-A and SnO_2_NPs-C, and 500 °C for SnO_2_NPs-D. The corresponding diffraction patterns are presented in Figure 4.

Figure 4 displays the XRD patterns of the calcined SnO_2_NPs, which match the tetragonal rutile-type SnO_2_ phase (space group P4_2_/mnm; JCPDS No. 41-1445), with lattice parameters a = b = 4.738 Å and c = 3.187 Å. The main diffraction peaks correspond to the following Miller indices: 26.6° (110), 33.9° (101), 38.0° (200), 51.8° (211), 54.8° (220), 57.9° (002), 61.9° (310), 64.9° (112), 66.0° (301), 78.7° (202), and 83.8° (321) [42]. The absence of peaks from other tin oxides or metallic tin confirms the high phase purity of the synthesized SnO_2_NPs. The high intensity and narrow full width at half maximum (FWHM) of the reflections indicate well-crystallized materials. The FWHM values increase slightly in the following order: SnO_2_NPs-B (calcined at 700 °C, Figure 4, trace B) < SnO_2_NPs-A (600 °C, Figure 4, trace A) < SnO_2_NPs-A’ (400 °C, Figure 4, trace A’) < SnO_2_NPs-C (600 °C, Figure 4, trace C) < SnO_2_NPs-D (500 °C, Figure 4, trace D), implying a gradual decrease in crystallite size.

For SnO_2_NPs-A calcined at 600 °C (Figure 4, trace A), the (101) diffraction is approximately 25% more intense than the (110) reflection, suggesting preferential growth along the (101) direction. In contrast, SnO_2_NPs-A’ (400 °C) and all green-synthesized samples, show dominant (110) reflection, indicating preferential exposure of the (110) surface. Crystallite sizes were estimated using the Debye–Scherrer equation, and the results are summarized in Table 1. As expected, crystallite size decreases gradually with decreasing calcination temperature [43].

**Figure 5:**
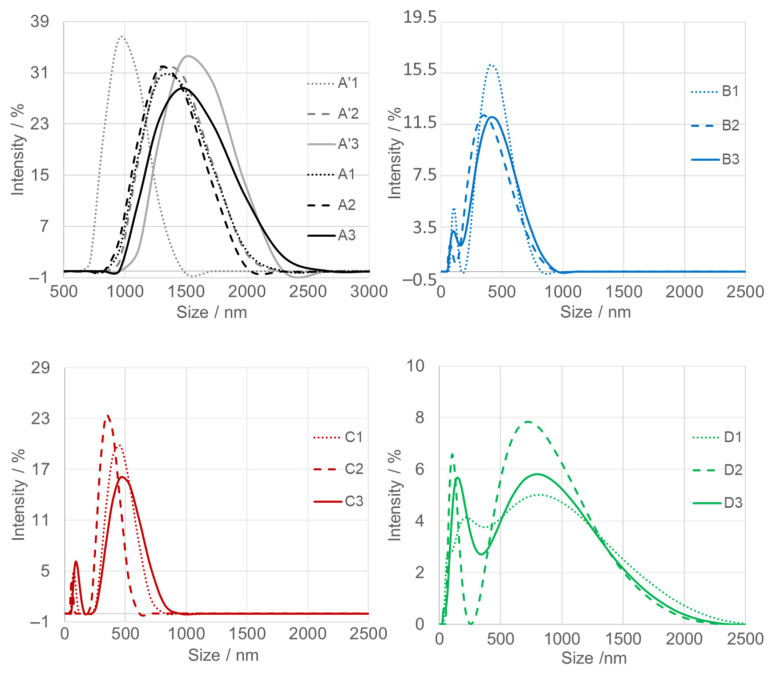
Dynamic light scattering (DLS) analysis of calcined SnO_2_ nanoparticles (SnO_2_NPs) synthesized via the wet-chemistry method (A, calcined at 400 °C and A’, at 600 °C), and via the green chemistry method using extracts from (B) rooibos leaves, (C) pomegranate seeds, and (D) kiwifruit peels.

Dynamic light scattering (DLS) analysis (Figure 5) was conducted in triplicate for each SnO_2_NPs suspension (dotted, dashed, and solid traces) to ensure the reproducibility of the particle size distribution profiles. Prior to measurement, all aqueous suspensions were sonicated; however, dispersions exhibited limited stability, leading to gradual sedimentation. Slight variations were observed between consecutive measurements. The first measurement, representing the “largest population,” is denoted as “1.” Over time, in stages two and three, the nanoparticles underwent gravitational settling, which resulted in a decrease in apparent hydrodynamic diameter. The average particle sizes estimated from DLS are summarized in Table 1.

**Figure 6:**
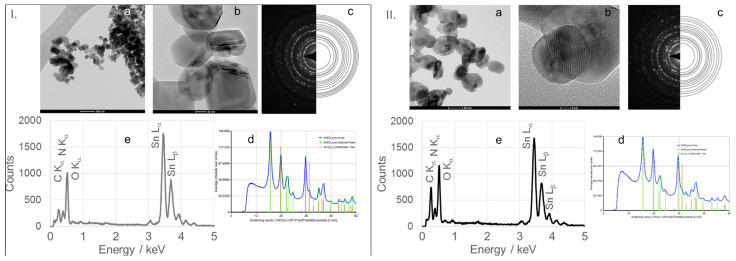
(**a**) TEM image, (**b**) high-resolution TEM (HRTEM) image, (**c**) selected area electron diffraction (SAED) pattern, (**d**) circularly averaged intensity from SAED as a function of the scattering vector, and (**e**) EDS analysis of SnO_2_ nanoparticles (SnO_2_NPs) synthesized via the wet-chemistry method and calcined at 400 °C (**I**) and at 600 °C (**II**).

**Figure 7:**
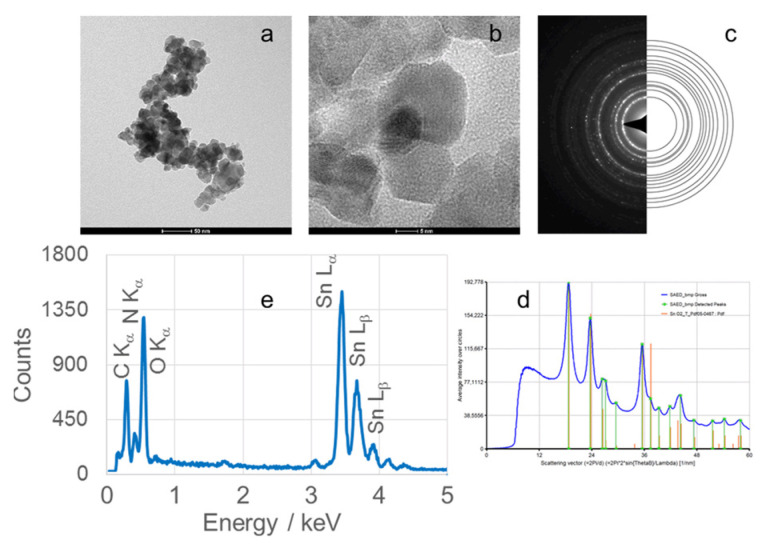
(**a**) TEM image, (**b**) high-resolution TEM (HRTEM) image, (**c**) selected area electron diffraction (SAED) pattern, (**d**) circularly averaged intensity from SAED as a function of the scattering vector, and (**e**) EDS analysis of SnO_2_ nanoparticles (SnO_2_NPs) synthesized using rooibos leaf extract and calcined at 700 °C.

**Figure 8:**
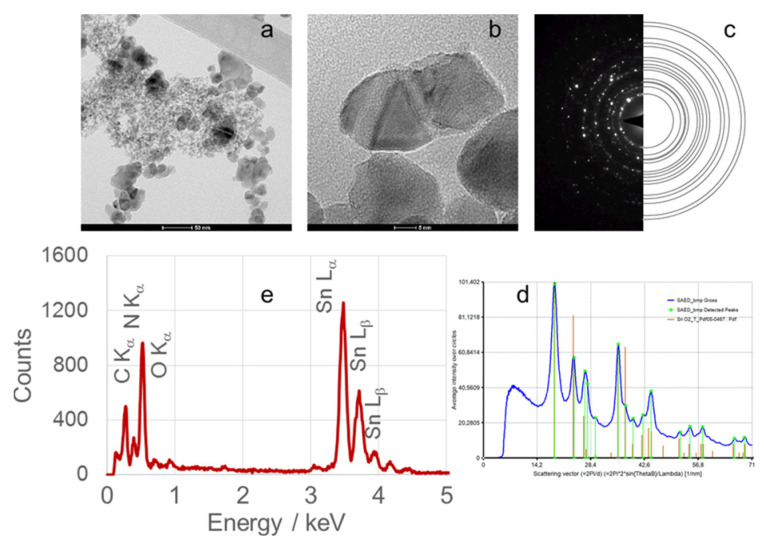
(**a**) TEM image, (**b**) high-resolution TEM (HRTEM) image, (**c**) selected area electron diffraction (SAED) pattern, (**d**) circularly averaged intensity from SAED as a function of the scattering vector, and (**e**) EDS analysis of SnO_2_ nanoparticles (SnO_2_NPs) synthesized using pomegranate seed extract and calcined at 600 °C.

**Figure 9:**
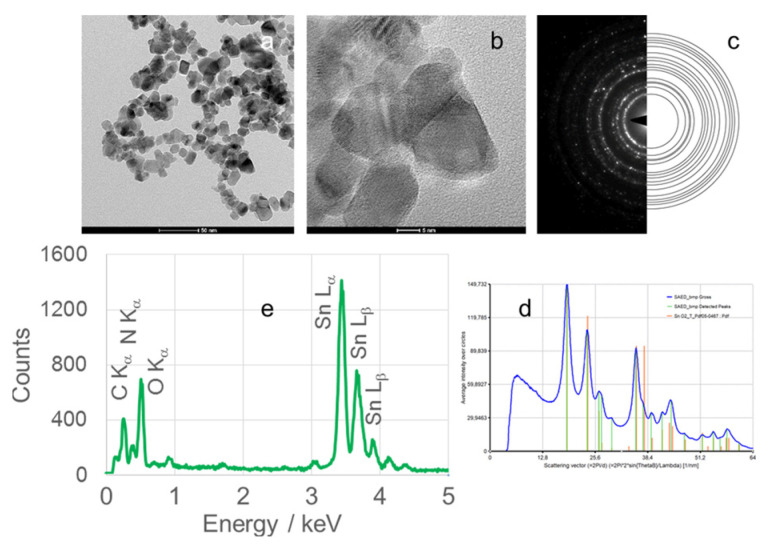
(**a**) TEM image, (**b**) high-resolution TEM (HRTEM) image, (**c**) selected area electron diffraction (SAED) pattern, (**d**) circularly averaged intensity from SAED as a function of the scattering vector, and (**e**) EDS analysis of SnO_2_ nanoparticles (SnO_2_NPs) synthesized using kiwifruit peel extract and calcined at 500 °C.

TEM micrographs, obtained at magnifications of 50 or 100 nm, of calcined SnO_2_NPs synthesized via the wet chemical route and green synthesis using rooibos leaves, pomegranate seeds, and kiwifruit peel extracts are shown in Figure 6 (images Ia and IIa), Figure 7, Figure 8 and Figure 9 (images a), respectively. These images reveal predominantly spherical nanoparticles, homogeneously distributed and forming agglomerates. Such aggregation is attributed to attractive van der Waals forces, electrostatic interactions (especially in high-ionic-strength or extreme pH environments) [44], and Ostwald ripening phenomena [45].

High-resolution TEM (HRTEM) images (Figure 6(Ib,IIb), Figure 7b, Figure 8b and Figure 9b) confirm the formation of well-defined spherical crystallites with clear lattice fringes, indicative of high crystallinity. The measured d-spacings between adjacent lattice planes were 0.26, 0.19, 0.17, 0.12, and 0.12 nm for SnO_2_NPs synthesized via the wet chemistry method (calcined at 400 °C and 600 °C) and with extracts from rooibos, pomegranate, and kiwifruit, respectively.

The selected area electron diffraction (SAED) patterns (Figure 6(Ic,IIc), Figure 7c, Figure 8c and Figure 9c) display continuous concentric rings, confirming the polycrystalline nature of the SnO_2_NPs. SAED patterns were converted into radial diffraction profiles (Figure 6(Id,IId), Figure 7d, Figure 8d and Figure 9d) using software that represents the circularly averaged diffraction intensity as a function of the scattering vector. Analysis of the intensity maxima corresponding to diffraction spots allowed for the calculation of d-spacing values, summarized in Table 2.

Energy-dispersive X-ray spectroscopy (EDS) profiles (Figure 6(Ie,IIe), Figure 7e, Figure 8e and Figure 9e) provide additional confirmation of SnO_2_ formation. All spectra exhibit four characteristic peaks in the 0.5–4.0 keV range [46]. Table 1 lists the corresponding energies for each oxide. Small peaks at approximately 0.26 keV (C Kα) and 0.39 keV (N Kα) were observed in all samples, attributed to residual gases (CO and N_2_) in the TEM chamber [47]. No additional peaks were detected, confirming the phase purity of the SnO_2_NPs.

The Sn and O atomic percentages reported in Table 1 show clear deviations from the ideal SnO_2_ stoichiometry (O:Sn = 2:1). Specifically, the measured values are: O = 48.22 at.%/Sn = 30.46 at.%, O = 42.99 at.%/Sn = 36.84 at.%, O = 57.33 at.%/Sn = 22.52 at.%, O = 54.75 at.%/Sn = 28.01 at.%, and O = 42.81 at.%/Sn = 39.87 at.%. These deviations are consistent with literature-reported atomic percentages obtained from EDS analyses of SnO_2_, which typically show a broad range depending on synthesis method, sample preparation, and measurement conditions. In particular, oxygen content in EDS studies can vary from ~50 to 81 at.% and tin from ~7 to 33 at.% [48,49,50,51], although highly stoichiometric commercial powders often exhibit O ≈ 80 at.% and Sn ≈ 19–20 at.% [48,49,50,51]. The discrepancies observed in Table 1 can therefore be attributed to factors such as the lower sensitivity of EDS to oxygen, the presence of surface contaminants or substrate effects, differences in quantification algorithms, and the limited probing depth of EDS compared to surface-sensitive techniques such as XPS. Consequently, deviations from the ideal O/Sn ratio of 2:1 are frequently reported and should be considered typical for EDS characterization of SnO_2_ nanoparticles.

Growing evidence indicates that the synthesis route plays a decisive role in determining the degree of non-stoichiometry in SnO_2_. In particular, green chemistry approaches—employing plant extracts as reducing, capping, and complexing agents—often produce materials that are more oxygen-deficient (SnO_2_^−^_x_) than those obtained by conventional routes. Plant extracts contain polyphenols, sugars, proteins, and other phytochemicals, which create locally oxygen-deficient environments during nucleation and crystallization. This promotes the formation of oxygen vacancies and partial reduction of Sn^4+^ to Sn^2+^, both of which are well-established indicators of non-stoichiometry [52,53]. Green-synthesized SnO_2_ nanoparticles frequently exhibit spectral signatures of vacancy-related defects (PL, EPR), mixed Sn^4+^/Sn^2+^ chemical states in XPS, and visible color tints associated with oxygen deficiency, which are often more pronounced than in conventionally synthesized reference materials [54,55,56]. Importantly, low-temperature wet-chemical methods (<150–200 °C)—including sol–gel and biosynthesis—do not provide sufficient thermal energy or oxygen mobility to fully oxidize tin to Sn^4+^, further stabilizing oxygen-deficient SnO_2_^−^_x_ phases [57].

At the same time, the extent of non-stoichiometry in SnO_2_ is not dictated solely by the synthesis route, but also by factors such as oxygen partial pressure, temperature, presence of reducing species, and post-treatment conditions. High-temperature treatments (>500–600 °C) in oxygen-rich atmospheres promote re-oxidation and vacancy annihilation, shifting the material toward stoichiometric SnO_2_, whereas low-temperature synthesis (≤100 °C) under limited oxygen availability favors stabilization of oxygen-deficient SnO_2_^−^_x_ [57,58,59]. Therefore, green-synthesized SnO_2_ nanoparticles and low-temperature wet-chemical routes converge mechanistically toward the same outcome: increased oxygen deficiency and higher deviation from ideal stoichiometry, driven by limited oxidation potential and restricted defect annealing—a trend consistently observed across studies on SnO_2_ defect chemistry [58,59].

**Figure 10:**
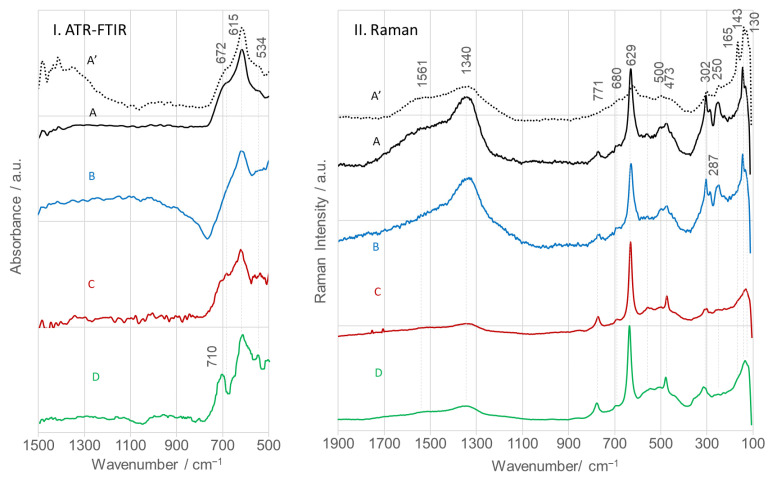
ATR-FTIR (**I**) and Raman (**II**) spectra of calcined SnO_2_ nanoparticles (SnO_2_NPs) synthesized via (A, calcined at 400 °C and A’, at 600 °C) the wet-chemistry method and the green chemistry method using extracts from (B) rooibos leaves, (C) pomegranate seeds, and (D) kiwifruit peels.

ATR-FTIR spectra of calcined SnO_2_NPs (Figure 10I) exhibit characteristic bands at 672, 615, and 534 cm^−1^, assigned to ν(O–Sn–O), ν(Sn–O), and ν(Sn–O, TO) modes, respectively [31]. Y. Zhang et al. alternatively assigned the 615 and 534 cm^−1^ bands to antisymmetric (E_u_, TO) and symmetric (A_2u_, TO) Sn–O–Sn vibrations [60]. The observed deviations in wavenumbers relative to literature values (618 and 477 cm^−1^) can be attributed to differences in particle size and morphology. All spectral features observed are consistent with IR bands reported for SnO_2_ synthesized via wet chemistry and calcined at 500 °C [30]. Discrepancies reported in other studies [61,62] likely result from differences in phase composition (monocrystal, powder, sol), defect density, stoichiometry, and morphology [63,64,65,66].

SnO_2_ with a tetragonal rutile structure (point group D_4h_^14^, space group P4_2_/mnm) contains two Sn^4+^ cations (coordination number 6) and four O^2−^ anions (coordination number 3) per unit cell [67]. Group theory predicts the following vibrational modes at the Γ point of the Brillouin zone: Γ = Γ^+^_1_(A_1g_) + Γ^+^_2_(A_2g_) + Γ^+^_3_(B_1g_) + Γ^+^_4_(B_2g_) + 2Γ^−^_5_(E_g_) + 2Γ^−^_1_(A_2_u) + 2Γ^−^_4_(B_1_u) + 4Γ^+^_5_(Eu). Among these, B_1g_, E_g_, A_1g_, and B_2g_ are Raman active [43,68].

Raman spectra (Figure 10II) display distinct peaks at 143, 473, 629, and 771 cm^−1^, corresponding to these modes [69]. The 629 and 771 cm^−1^ bands arise from Sn–O stretching vibrations. In the A_1_g mode, two bonds shorten while the remaining four expand, whereas in the B_2_g mode, all six Sn–O bonds vibrate in phase. The 629 cm^−1^ feature is intense in highly crystalline nanoparticles (>75–100 nm) but broadens and downshifts as particle size decreases [69]. In contrast, the 473 and 771 cm^−1^ bands increase in relative intensity with decreasing particle size, consistent with the present data.

Additional Raman bands at 143 (A_2_g) and 165 cm^−1^ (B_1_g) were observed for SnO_2_NPs synthesized by the wet chemistry route and with rooibos extract. These are attributed to displacement of O^2−^ ions from the diagonal plane, likely caused by rotation of the O–Sn–O bond around the Sn^4+^ axis. The 165 cm^−1^ mode, detected only in SnO_2_NPs-A calcined at 400 °C, is associated with oxygen vacancies and local disorder in the (200) planes [65]. An additional IR-active band at 302 cm^−1^ was detected in all spectra [29]. The SnO_2_NPs-A and SnO_2_NPs-B samples exhibited medium-intensity 302 cm^−1^ bands, while weaker responses were noted in the other samples. The presence of this feature confirms reduced nanoparticle size and surface defects, such as oxygen vacancies, which significantly influence their electronic and catalytic properties [70].

**Figure 11:**
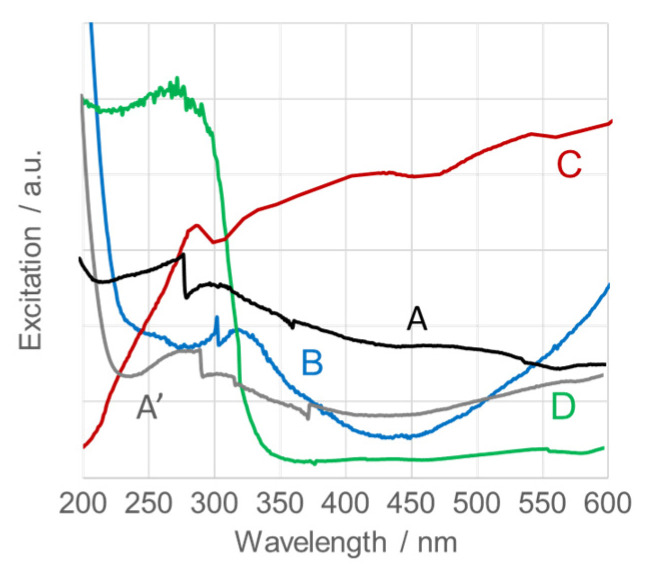
UV-Vis spectra of calcined SnO_2_ nanoparticles (SnO_2_NPs) synthesized via (A, calcined at 400 °C and A’, at 600 °C) the wet-chemistry method and the green chemistry method using extracts from (B) rooibos leaves, (C) pomegranate seeds, and (D) kiwifruit peels.

Figure 11 shows the UV–Vis spectra of calcined SnO_2_NPs in the 200–600 nm range. The absorption edge, corresponding to bandgap transitions, appears in the UV region. The absorption maxima and corresponding bandgap energies, calculated using Tauc’s relation, are summarized in Table 1 [71]. The observed bandgaps exhibit a blue shift compared to the theoretical value for bulk tetragonal rutile SnO_2_ (E_g_ = 3.64 eV) and previously reported 2D nanosheets (λ_abs_ = 280 nm) and SnO_2_ nanoparticles (λ_abs_ = 260–285 nm, 2–5 nm in diameter) synthesized by solvothermal processes [57,72]. A red shift relative to hydrothermally prepared SnO_2_NPs (λ_abs_ = 370 nm, 11–12 nm) [73] is also evident. These results agree well with reported data for SnO_2_ (λ_abs_ ≈ 325 nm, 10–15 nm) [74]. The observed blue shift with decreasing nanoparticle size confirms the quantum confinement effect, demonstrating that the bandgap energy of calcined SnO_2_NPs increases inversely with particle size.

### 3.2. Photocatalytic Activity

**Figure 12:**
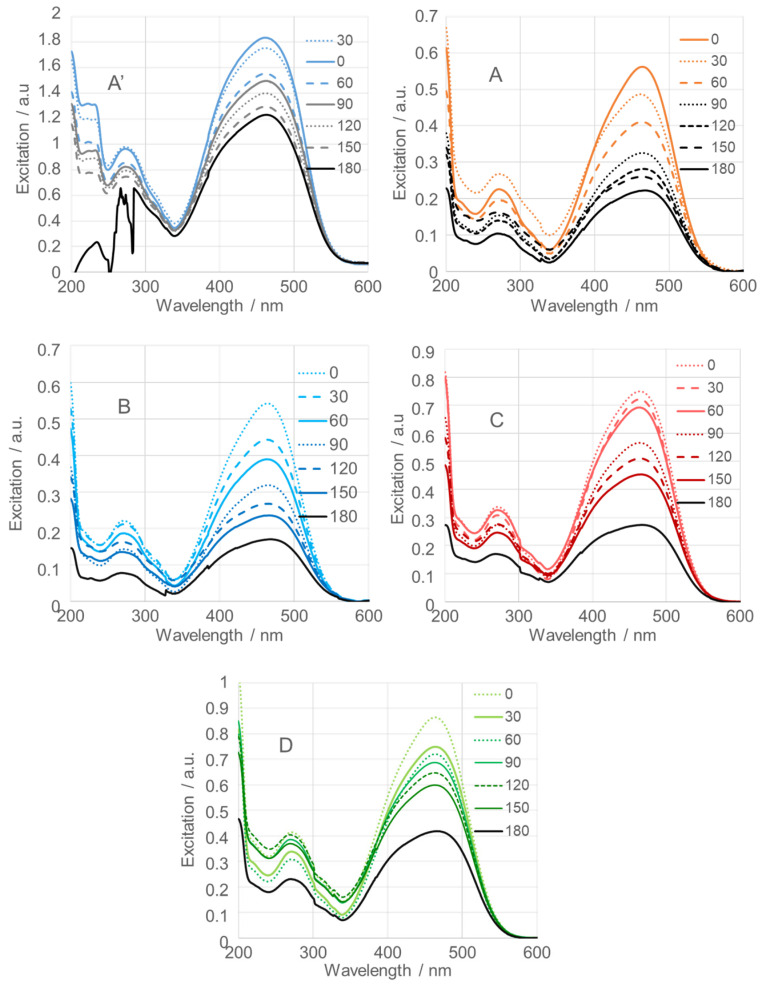
Photodegradation efficiency of calcined SnO_2_ nanoparticles (SnO_2_NPs) synthesized via the wet-chemistry method (**A**, calcined at 400 °C and **A’**, at 600 °C) and the green chemistry method using extracts from (**B**) rooibos leaves, (**C**) pomegranate seeds, and (**D**) kiwifruit peels.

Figure 12 presents the time-dependent excitation spectra of methyl orange (MeO), a common synthetic azo dye. MeO was used to evaluate the photocatalytic activity of SnO_2_NPs under 365 nm light irradiation. A mixture containing SnO_2_NPs, MeO, and distilled water in a molar ratio of approximately 1:4.5:8 was irradiated. Spectra were recorded every 30 min over a 180 min irradiation period, starting immediately after mixing (0 min).

As shown in the figure, SnO_2_NPs obtained using rooibos leaf extract (Figure 12B), with an average crystallite size of 71.1 nm, exhibited the highest photocatalytic efficiency, degrading 68% of MeO within 180 min. In contrast, SnO_2_NPs synthesized using kiwifruit extract, with a crystallite size of 18.2 nm, achieved a degradation efficiency of 52% (Figure 12D), while those prepared from pomegranate seed extract, with a crystallite size of 27.9 nm, showed a 63% degradation efficiency (Figure 12C).

SnO_2_NPs prepared by the wet chemical method (Figure 12A, calcined at 600 °C, and Figure 12A’, calcined at 400 °C) displayed degradation efficiencies of 60% and 33%, respectively, corresponding to crystallite sizes of 43.6 and 37.4 nm. Table 1 summarizes the photocatalytic efficiencies of all investigated SnO_2_NPs.

### 3.3. Adsorption Monitored by SERS

Tin (Sn) is generally considered an inactive transition metal in surface-enhanced Raman spectroscopy (SERS). To effectively amplify the Raman signal on Sn and SnO_2_ nanostructures, SERS experiments typically require ultraviolet excitation or Sn/SnO_2_ nanostructures coated or doped by noble metals. This approach exploits the enhanced electromagnetic field at the rough nanoscale surface of noble metals. The enhanced field of the noble metal surface penetrates the interface between the transition metal and the surrounding medium, where SERS excitation occurs.

Previous studies have employed gold-coated SnO_2_ nanoparticles to monitor the adsorption of ciprofloxacin [75] and pyridine [76]; Cu-doped SnO_2_–NiO heterostructures have been used to detect volatile organic compounds [77]; and Ag-coated SnO_2_NPs have been applied for rhodamine 6G sensing [78]. In contrast, two reports demonstrated the use of pure SnO_2_ nanoparticles as SERS-active substrates. Jiang et al. showed that octahedral SnO_2_ nanoparticles effectively monitored 4-mercaptobenzoic acid (4-MBA) and 4-nitrobenzenethiol (4-NBT) adsorbed on their surfaces [79], while Hou et al. employed sol-hydrothermally prepared SnO_2_ nanoparticles as SERS substrates for 4-MBA detection [80]. Notably, both 4-MBA and 4-NBT are high-symmetry aromatic molecules containing lone-pair-rich functional groups, which exhibit strong affinity toward metallic surfaces. The present study represents the third reported example of using SnO_2_NPs as a SERS substrate, but the first involving biologically active, low-symmetry molecules.

**Figure 13:**
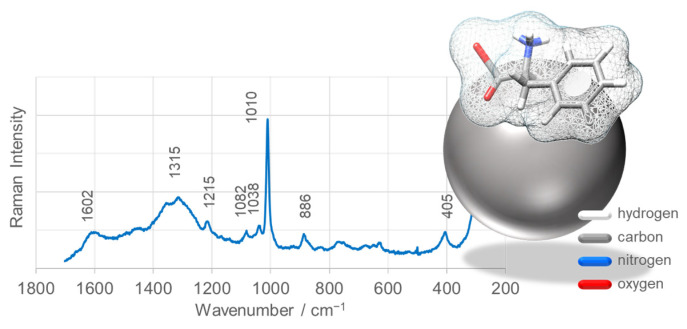
SERS spectra of L-phenylalanine (L-Phe) adsorbed on the surface of SnO_2_ nanoparticles (SnO_2_NPs) synthesized using rooibos leaf extract. Insets: Proposed adsorption geometry of L-Phe on the SnO_2_NP surface.

Figure 13 presents the SERS spectrum of L-phenylalanine (L-Phe) adsorbed onto SnO_2_NPs-B, synthesized using rooibos leaf extract and calcined at 700 °C. These nanoparticles were selected due to their superior photocatalytic efficiency in methyl orange degradation. The spectrum displays enhanced bands at 1602 (ν_8_a and ν_8_b), 1315 (ν_14_), 1215 (ν_7_a), 1082 (ν_18_b), 1038 (ν_18_a), 1010 (ν_12_), and 886 cm^−1^ (ν_10_a), corresponding to characteristic vibrations of the aromatic ring of L-Phe [81,82,83]. These results indicate that L-Phe predominantly interacts with the SnO_2_NP surface via its phenyl ring.

Detailed analysis of band intensities, wavenumbers, and full width at half maximum (FWHM), particularly the ν_12_ mode, provides insights into the orientation of the aromatic ring on the nanoparticle surface. The ν_12_ band is shifted to lower frequencies by 8 cm^−1^, broadened (ΔFWHM = +4 cm^−1^), and less enhanced compared with the corresponding Raman band, suggesting a tilted orientation of the phenyl ring relative to the SnO_2_NP surface. Additionally, a broad feature with two maxima at 1315 and 1360 cm^−1^ is observed, corresponding to ν(CH_2_) and ν_s_(COO_2_) vibrations, further supporting a tilted ring configuration in which the aliphatic fragment and carboxyl group are directed toward the SnO_2_NP surface (Figure 13).

These observations reinforce the concept that the adsorption behavior and molecular orientation on a nanoparticle surface are strongly substrate-dependent. For instance, on Zn and ZnO nanoparticles, L-phenylalanine interacts primarily via its aromatic ring [84]; on AgNPs, both the aromatic ring and carboxyl group participate [82]; on MgO nanoparticles, the aromatic ring and amino group are involved [85]; and on AuNPs or iron oxide nanoparticles (α-Fe and γ-Fe_2_O_3_), simultaneous coordination via the aromatic ring, amino, and carboxyl groups has been reported [86,87].

The SERS enhancement factor (EF), quantifying the substrate’s effectiveness, was calculated for the SnO_2_NPs and compared with previously reported values for other SERS-active materials. The EF for SnO_2_NPs reached up to 10^2^, comparable to MgO, Zn, Pt, and Fe substrates, yet lower than those for AgNPs and Au@SiO_2_ (10^6^), AuNPs (10^5^), Cu and Ti (10^4^), and ZnO-, CuO-, Cu_2_O-, TiO_2_-, and γ-Fe_2_O_3_-based nanostructures (10^3^) [82,84,85,86,87,88,89].

## 4. Conclusions

This study reports the synthesis, structural characterization, and functional evaluation of four types of tin dioxide nanoparticles (SnO_2_NPs). One sample was obtained using a conventional chemical method, while the other three were synthesized via an environmentally friendly green chemistry approach employing reducing agents naturally present in plant extracts. Three easily accessible plants—pomegranate seeds, rooibos leaves, and kiwi fruit peels—were selected as sources of phytochemicals responsible for nanoparticle formation.

The crystalline nature of SnO_2_NPs plays a decisive role in determining their stability, cytotoxicity, and biocompatibility—parameters that are crucial for biomedical applications. Therefore, X-ray diffraction (XRD) analysis was employed to determine the crystalline structure of the as-prepared nanoparticles. The results indicated that the non-calcined SnO_2_NPs were predominantly amorphous, which necessitated thermal treatment. The calcination temperature for each sample was selected based on thermogravimetric (TGA) analysis. XRD patterns obtained after calcination revealed the preferred crystallite growth orientations: (110) for SnO_2_NPs-A synthesized by the wet-chemical method and calcined at 600 °C, and (101) for SnO_2_NPs-A’ calcined at 400 °C, as well as for samples synthesized using plant extracts. The average crystallite size ranged from 18.2 to 71.1 nm.

Transmission electron microscopy (TEM) and dynamic light scattering (DLS) analyses showed a relatively homogeneous morphology consisting of agglomerated spherical particles and a controlled size distribution, with the mean particle size variation in suspension not exceeding 19% (SnO_2_NPs-A’), 13% (SnO_2_NPs-B), 9% (SnO_2_NPs-C), and 3% (SnO_2_NPs-A and SnO_2_NPs-D). Energy-dispersive X-ray spectroscopy (EDS) confirmed the elemental composition and the absence of toxic impurities, further supporting their potential biocompatibility.

UV–Vis spectra revealed that the synthesized SnO_2_NPs exhibit reduced optical bandgap values (3.81–4.59 eV) compared to bulk SnO_2_, which enhances their surface reactivity and potential osseointegration. Raman and ATR-FTIR analyses confirmed the formation of non-stoichiometric oxides containing oxygen vacancy defects, which may contribute to their photocatalytic and sensing performance.

Photocatalytic tests demonstrated that SnO_2_NPs synthesized using rooibos extract (SnO_2_NPs-B) showed the highest activity, degrading 68% of methyl orange under UV irradiation within 180 min. Owing to their superior photocatalytic efficiency, SnO_2_NPs-B were further employed as a SERS-active substrate for studying the adsorption of L-phenylalanine (L-Phe). The obtained SERS spectra confirmed the sensitivity of these nanoparticles toward biologically active molecules and their ability to probe molecular interactions at the surface of bone scaffold materials.

Overall, this work provides the third reported example of using pure SnO_2_ nanoparticles as a SERS substrate, and the first demonstration of their applicability for biologically active compounds and molecules of low symmetry. The findings highlight the potential of green-synthesized SnO_2_NPs as multifunctional materials—combining photocatalytic, sensing, and biocompatible properties—which can be further developed for biomedical and environmental applications.

## Figures and Tables

**Table 1:** Surface analysis results of calcined SnO_2_ nanoparticles (SnO_2_NPs).

SnO_2_NPs	XRD			DLS Average Particle Size in Suspension [nm]	EDS			UV-Vis		
	Planes (hkl)	2ϴ [°]	Crystallite Size [nm]		[keV]		Atomic Content [%]	λ_abs_ [nm]	E_g_ [eV]	Catalytic Efficiency [%]
SnO_2_NPs-A’ (wet chemistry, 400 °C)	110	26.6274	42.78	1652	0.51	O K_α_	O: 48.22	291	4.26	33.4
	101	33.9275	32.04		3.44 3.68 3.91	Sn L_α_ Sn L_β_ Sn L_β_	Sn: 30.46			
SnO_2_NPs-A (wet chemistry, 600 °C)	110	26.6371	42.91	1859	0.52	O K_α_	O: 42.99	301	4.12	60.5
	101	33.9374	44.30		3.44 3.67 3.91	Sn L_α_ Sn L_β_ Sn L_β_	Sn: 36.84			
SnO_2_NPs-B (rooibos, 700 °C)	110	26.6368	76.35	383	0.51	O K_α_	O: 57.33	325	3.81	68.6
	101	33.963	65.90		3.45 3.67 3.91	Sn L_α_ Sn L_β_ Sn L_β_	Sn: 22.52			
SnO_2_NPs-C (pomegranate, 600 °C)	110	26.6816	26.99	497	0.52	O K_α_	O: 54.75	283	4.38	63.5
	101	34.0018	28.92		3.45 3.69 3.91	Sn L_α_ Sn L_β_ Sn L_β_	Sn: 28.01			
SnO_2_NPs-D (kiwifruit, 500 °C)	110	26.6515	17.13	263	0.51	O K_α_	O: 42.81	270	4.59	52.1
	101	33.9552	19.30		3.44 3.68 3.90	Sn L_α_ Sn L_β_ Sn L_β_	Sn: 39.87			

**Table 2:** d-spacing values of SnO_2_ nanoparticles (SnO_2_NPs) determined from circularly averaged intensity in SAED as a function of the scattering vector length.

SnO_2_NPs-A’ (Wet Chemistry, 400 °C)		SnO_2_NPs-A (Wet Chemistry, 600 °C)		SnO_2_NPs-B (Rooibos, 700 °C)		SnO_2_NPs-C (Pomegranate, 600 °C)		SnO_2_NPs-D (Kiwifruit, 500 °C)	
R [pix]	d [Å]	R [pix]	d [Å]	R [pix]	d [Å]	R [pix]	d [Å]	R [pix]	d [Å]
250	3.342	252	3.348	252	3.363	253	3.329	252	3.362
317	2.633	318	2.654	319	2.658	321	2.623	319	2.656
353	2.365	354	2.385	355	2.385	360	2.342	357	2.376
363	2.302	365	2.313	366	2.314	369	2.284	367	2.314
395	2.115	397	2.127	398	2.127	397	2.122	400	2.12
475	1.757	478	1.767	749	1.77	479	1.762	479	1.77
500	1.67	504	1.675	504	1.681	504	1.672	503	1.687
526	1.589	529	1.597	530	1.598	532	1.586	530	1.6
560	1.492	562	1.502	564	1.502	567	1.489	566	1.5
593	1.41	594	1.423	597	1.42	596	1.416	595	1.425
637	1.312	638	1.324	638	1.328	700	1.205	639	1.328
690	1.211	695	1.215	696	1.217	733	1.15	698	1.215
706	1.183	733	1.153	732	1.157	779	1.083	734	1.155
727	1.149	781	1.082	782	1.084	892	0.946	756	1.122
750	1.113					928	0.909	779	1.089
775	1.078							818	1.037

## Data Availability

Data available upon request (proniewi@agh.edu.pl).

## References

[1] Karmaoui M., Jorge A.B., McMillan P.F., Aliev A.E., Pullar R.C., Labrincha J.A., Tobaldi D.M. (2018). One-Step Synthesis, Structure, and Band Gap Properties of SnO_2_ Nanoparticles Made by a Low Temperature Nonaqueous Sol–Gel Technique. ACS Omega.

[2] Gu F., Wang S.F., Lu M.K., Zhou G.J., Xu D., Yuan D.R. (2004). Photoluminescence Properties of SnO_2_ Nanoparticles Synthesized by Sol-Gel Method. J. Phys. Chem. B.

[3] Kharbanda J., Priya R. (2022). Synthesis and Applications of Tin Oxide Nanoparticles: An Overview. Mater. Today Proc..

[4] Sharma V., Orlandi M.O. (2020). Optical Properties of Tin Oxide Nanomaterials. Tin Oxide Materials: Synthesis, Properties, and Applications.

[5] Zhang J., Su P., Chen H., Qiao M., Yang B., Zhao X. (2023). Impact of Reactive Oxygen Species on Cell Activity and Structural Integrity of Gram-Positive and Gram-Negative Bacteria in Electrochemical Disinfection System. Chem. Eng..

[6] Giridasappa A., Shareef M.I., Gopinath S.M., Rangappa D., Shivaramu P., Sabbanahalli C. (2023). Synthesis, Antioxidant, Bactericidal and Antihemolytic Activity of Al_2_O_3_ and SnO_2_ Nanoparticles. Proc. Natl. Acad. Sci. India Sect. B Biol. Sci..

[7] Michalec K., Mozgawa B., Kusior A., Pietrzyk P., Sojka Z., Radecka M. (2024). Tunable Generation of Reactive Oxygen Species in SnO_2_/SnS_2_ Nanostructures: Mechanistic Insights into Indigo Carmine Photodegradation. Phys. Chem. Chem. Phys..

[8] Masuda Y. (2022). Recent Advances in SnO_2_ Nanostructure Based Gas Sensors. Sens. Actuators B Chem..

[9] Dar M.A., Neelamegan H., Priyadharshini V.J., Ahamed S.R., Arularasan P., Mishra M., Rather A.A. (2024). Enhancing Superca-pacitor, Photovoltaic and Magnetic Properties of SnO_2_ Nanoparticles Doped with Cu and Zn Ions. Bull. Mater. Sci..

[10] Romero D.G., Di Mario L., Yan F., Ibarra-Barreno C.M., Mutalik S., Protesescu L., Rudolf P., Loi M.A. (2023). Understanding the Surface Chemistry of SnO_2_ Nanoparticles for High Performance and Stable Organic Solar Cells. Adv. Funct. Mater..

[11] Wang X., Song Y., Zhou L., Yang Q. (2023). Preparation and Performance of SnO_2_ Nanoparticles Encapsulated in Carbon Nanofibers as Anodes for Lithium-Ion Battery. Mater. Lett..

[12] Din S.U., Kiani S.H., Haq S., Ahmad P., Khandaker M.U., Faruque M.R.I., Idris A.M., Sayyed M.I. (2022). Bio-Synthesized Tin Oxide Nanoparticles: Structural, Optical, and Biological Studies. Crystals.

[13] Rehman S., Asiri S.M., Khan F.A., Jermy B.R., Khan H., Akhtar S., Jindan R.A., Khan K.M., Qurashi A. (2019). Biocompatible Tin Oxide Nanoparticles: Synthesis, Antibacterial, Anticandidal and Cytotoxic Activities. Chem. Select.

[14] Prasad R.G.S.V., Phani A.R., Rao K.N., Kumar R.R., Prasad S., Prabhakara G., Sheeja M.S., Salins C.P., Endrino J.L., Raju D.B. (2015). Biocompatible and Antibacterial SnO_2_ Nanowire Films Synthesized by E-Beam Evaporation Method. J. Biomed. Nanotechnol..

[15] Schwartz V.B., Thetiot F., Ritz S., Putz S., Choritz L., Lappas A., Forch R., Landfester K., Jonas U. (2012). Antibacterial Surface Coatings from Zinc Oxide Nanoparticles Embedded in Poly(N-Isopropylacrylamide) Hydrogel Surface Layers. Adv. Funct. Mater..

[16] Sagadevan S., Lett J.A., Fatimah I., Lokanathan Y., Léonard E., Oh W.C., Hossain M.A.M., Johan M.R. (2021). Current Trends in the Green Syntheses of Tin Oxide Nanoparticles and Their Biomedical Applications. Mater. Res. Express.

[17] Zhou R., Li Y.L., Li M., Cao J., Cheng J., Wei D., Li B., Wang Y., Jia D., Jiang B. (2024). Electrical Responsive Coating with a Multilayered TiO_2_–SnO_2_–RuO_2_ Heterostructure on Ti for Controlling Antibacterial Ability and Improving Osseointegration. ACS Appl. Mater. Interfaces.

[18] Hsu S.-H., Liao H.-T., Chen R.-S., Chiu S.-C., Tsai F.-Y., Lee M.S., Hu C.-Y., Tseng W.-Y. (2023). The Influence on Surface Characteristic and Biocompatibility of Nano-SnO_2_–Modified Titanium Implant Material Using Atomic Layer Deposition Technique. J. Formos. Med. Assoc..

[19] Díez-Pascual A.M., Díez-Vicente A. (2017). Antibacterial SnO_2_ Nanorods as Efficient Fillers of Poly(Propylene Fumarate-Co-Ethylene Glycol) Biomaterials. Mater. Sci. Eng. C.

[20] Fakhria A., Behrouzb S. (2015). Synthesis, Photocatalytic and Antimicrobial Properties of SnO_2_, SnS_2_ and SnO_2_/SnS_2_ Nanostructure. J. Photochem. Photobiol. B.

[21] Liang X., Dai R., Wang Q., Zhang B. (2023). Antibacterial Activity of SnO_2_ in Visible Light Enhanced by Erbium–Cobalt Co-Doping. Colloids Surf. A.

[22] Bamagous G.A., Ibrahim I.A.A., Alzahrani A.R., Shahid I., Shahzad N., Falemban A.H., Alanazi I.M.M., Hussein-Al-Ali S.H., Arulselvan P., Thangavelu I. (2025). Multifunctional SnO_2_–Chitosan–D-Carvone Nanocomposite: A Promising Antimicrobial, Anticancer, and Antioxidant Agent for Biomedical Applications. J. Inorg. Organomet. Polym. Mater..

[23] Guo Y., Zhao Y., Zhao X., Song S., Qian B. (2021). Exploring the Anticancer Effects of Tin Oxide Nanoparticles Synthesized by Pulsed Laser Ablation Technique against Breast Cancer Cell Line Through Downregulation of PI3K/AKT/mTOR Signaling Pathway. Arab. J. Chem..

[24] Ruan R., Chen R., Yu H. (2024). Synthesized Tin Oxide Nanoparticles Promote Apoptosis in Human Osteosarcoma Cells. Arab. J. Chem..

[25] Hanna D.H., Saad G. (2021). Induction of Mitochondria Mediated Apoptosis in Human Ovarian Cancer Cells by Folic Acid Coated Tin Oxide Nanoparticles. PLoS ONE.

[26] Ahamed M., Akhtar M.J., Majeed Khan M.A., Alhadlaq H.A. (2018). Oxidative Stress Mediated Cytotoxicity of Tin (IV) Oxide (SnO_2_) Nanoparticles in Human Breast Cancer (MCF-7) Cells. Colloids Surf. B.

[27] Ahmadabada L.E., Kalantaria F.S., Liu H., Hasan A., Gamasaee N.A., Edis Z., Attar F., Ale-Ebrahim M., Rouholla F., Babadaei M.M.N. (2021). Hydrothermal Method-Based Synthesized Tin Oxide Nanoparticles: Albumin Binding and Antiproliferative Activity Against K562 Cells. Mater. Sci. Eng. C.

[28] Ma J., Zhao M., Zhang C., Wu X., Yang G. (2020). Synthesis of L. Acidissima Mediated Tin Oxide Nanoparticles for Cervical Car-cinoma Treatment in Nursing Care. J. Drug Deliv. Sci. Technol..

[29] Wan W., Li Y., Ren X., Zhao Y., Gao F., Zhao H. (2018). 2D SnO_2_ Nanosheets: Synthesis, Characterization, Structures, and Excellent Sensing Performance to Ethylene Glycol. Nanomaterials.

[30] Amalric-Popescu D., Bozon-Verduraz F. (2001). Infrared Studies on SnO_2_ and Pd/SnO. Catal. Today.

[31] Ghiotti G., Chiorino A., Boccuzzi F. (1989). Infrared study of surface chemistry and electronic effects of different atmospheres on SnO_2_. Sens. Actuators B.

[32] Ma X.L., Li Y., Zhu Y.L. (2003). Growth mode of the SnO_2_ nanobelts synthesized by rapid oxidation. Chem. Phys. Lett..

[33] Gao C., Yuan S., Cao B., Yu J. (2017). SnO_2_ nanotube arrays grown via an in situ template-etching strategy for effective and stable perovskite solar cells. Chem. Eng. J..

[34] Li Y., Peng R., Xiu X., Zheng X., Zhang X., Zha G. (2011). Growth of SnO_2_ nanoparticles via thermal evaporation method. Superlattices Microstruct..

[35] Li X.-B., Wang X.-W., Shen Q., Zheng J., Liu W.-H., Zhao H., Yang F., Yang H.-Q. (2013). Controllable Low-Temperature Chemical Vapor Deposition Growth and Morphology Dependent Field Emission Property of SnO_2_ Nanocone Arrays with Different Mor-phologies. ACS Appl. Mater. Interfaces.

[36] Patel G.H., Chakia S.H., Kannaujiya R.M., Parekh Z.R., Hirpara A.B., Khimani A.J., Deshpande M.P. (2021). Sol–gel synthesis and thermal characterization of SnO_2_ nanoparticles. Phys. B.

[37] Council Directive 1999/31/EC of 26 April 1999 on the Landfill of Waste. https://eur-lex.europa.eu/eli/dir/1999/31/oj?utm_source=chatgpt.com.

[38] Waste Framework Directive legislation. https://echa.europa.eu/pl/wfd-legislation?utm_source=chatgpt.com.

[39] Marakatti V.S., Shanbhag G.V., Halgeri A.B. (2013). Condensation reactions assisted by acidic hydrogen bonded hydroxyl groups in solid tin(II) hydroxychloride. RSC Adv..

[40] Ocafia M., Serna C.J., Matijevic E. (1995). Formation of “monodispersed” SnO_2_ powders of various morphologies. Colloid. Polym. Sci..

[41] Séby F., Potin-Gautier M., Giffaut E., Donard O.F.X. (2001). A critical review of thermodynamic data for inorganic tin species. Geochim. Cosmochim. Acta.

[42] Patil G.E., Kajale D.D., Gaikwad V.B., Jain G.H. (2012). Preparation and characterization of SnO_2_ nanoparticles by hydrothermal route. Int. Nano Lett..

[43] Khanam J., Hasan R., Biswas B., Ahmed F., Mostofa S., Akhtar U.S., Hossain K., Quddus S., Ahmed S., Sharmin N. (2024). Effect of low temperature calcination on microstructure of hematite nanoparticles synthesized from waste iron source. Heliyon.

[44] Baalousha M. (2009). Aggregation and disaggregation of iron oxide nanoparticles: Influence of particle concentration, pH and natural organic matter. Sci. Total Environ..

[45] Behrens M.A., Franzén A., Carlert S., Skantze U., Lindfors L., Olsson U. (2025). On the Ostwald ripening of crystalline and amorphous nanoparticles. Soft Matter.

[46] Doubi Y., Hartiti B., Batan A., Siadat M., Labrim H., Tahiri M., Kotbi A., Thevenin P., Jouiad M. (2025). Controlled SnO_2_ nanostructures for enhanced sensing of hydrogen sulfide and nitrogen dioxide. Sens. Actuators B.

[47] Hettler S., Dries M., Hermann P., Obermair M., Gerthsen D., Malac M. (2017). Carbon contamination in scanning transmission electron microscopy and its impact on phase-plate applications. Micron.

[48] Izydorczyk W., Izydorczyk J. (2021). Structure, Surface Morphology, Chemical Composition, and Sensing Properties of SnO_2_ Thin Films in an Oxidizing Atmosphere. Sensors.

[49] Lee M.H., Mirzaei A., Kim H.W., Kim S.S. (2024). Optimization of Deposition Parameters of SnO_2_ Particles on Tubular Alumina Substrate for H_2_ Gas Sensing. Appl. Sci..

[50] Rahman M.d.T., Ahmed Z., Islam M.d.J., Kamaruzzaman, Khatun M.d.T., Gafur M.d.A., Bashar M.d.S., Alam M.d.M. (2021). Comparative Study of Structural, Optical and Electrical Properties of SnO_2_ Thin Film Growth via CBD, Drop-Cast and Dip-Coating Methods. Mater. Sci. Appl..

[51] Manjula N., Selvan G., Perumalsamy R., Thirumamagal R., Ayeshamariam A., Jayachandran M. (2016). Synthesis, Structural and Electrical Characterizations of SnO_2_ Nanoparticles. Int. J. Nanoelectron. Mater..

[52] Gebreslassie Y.T., Gebretnsae H.G. (2021). Green and Cost-Effective Synthesis of Tin Oxide Nanoparticles: A Review on the Synthesis Methodologies, Mechanism of Formation, and Their Potential Applications. Nano Res. Lett..

[53] Quintero González E., Lugo Medina E., Quevedo Robles R.V., Garrafa Gálvez H.E., Baez Lopez Y.A., Vargas Viveros E., Aguilera Molina F., Vilchis Nestor A.R., Luque Morales P.A. (2022). A Study of the Optical and Structural Properties of SnO_2_ Nanoparticles Synthesized with *Tilia cordata* Applied in Methylene Blue Degradation. Symmetry.

[54] Tasisa Y.E., Sarma T.K., Sahu T.K., Sahu T.K., Krishnaraj R. (2024). Phytosynthesis and characterization of tin-oxide nanoparticles (SnO_2_-NPs) from *Croton macrostachyus* leaf extract and its application under visible light photocatalytic activities. Sci. Rep..

[55] Chang S.-S., Yoon S.O., Park H.J. (2005). Characteristics of SnO_2_ Annealed in Reducing Atmosphere. Ceram. Internat..

[56] Liaqat F., Vosqa U.t., Khan F., Haleem A., Shaik M.R., Siddiqui M.R.H., Khan M. (2023). Light-Driven Catalytic Activity of Green-Synthesized SnO_2_/WO_3−x_ Hetero-Nanostructures. ACS Omega.

[57] Osuntokun J., Onwudiwe D.C., Ebenso E.E. (2017). Biosynthesis and Photocatalytic Properties of SnO_2_ Nanoparticles Prepared Using Aqueous Extract of Cauliflower. J. Clust. Sci..

[58] Alagdal I.A., West A.R. (2015). Oxygen Non-Stoichiometry, Conductivity and Gas Sensor Response of SnO_2_ Pellets. J. Mater. Chem. A.

[59] Woo S.W., Seo H.B., Choi J., Bae B.S., Yun E.-J. (2019). Effects of Process Parameters on the Properties of Sputter-Deposited Tin Oxide Thin Films. J. Nanosci. Nanotechnol..

[60] Zhang Y., Li L., Zheng J., Li Q., Zuo Y., Yang E., Li G. (2015). Two-Step Grain-Growth Kinetics of Sub-7 nm SnO_2_ Nanocrystals under Hydrothermal Conditions. J. Phys. Chem. C.

[61] Haridas D., Chowdhuri A., Sreenivas K., Gupta V. (2011). Effect of Thickness of Platinum Catalyst Clusters on Response of SnO_2_ Thin Film Sensor for LPG. Sens. Actuators B Chem..

[62] Juliasih N., Buchari, Noviandri I. (2017). Application of SnO_2_ Nanoparticle as Sulfide Gas Sensor Using UV/VIS/NIR Spectrophotometer. IOP Conf. Ser. Mater. Sci. Eng..

[63] Hollins P. (1992). The Influence of Surface Defects on the Infrared Spectra of Adsorbed Species. Surf. Sci. Rep..

[64] Genzel L., Martin T.P. (1973). Infrared Absorption by Surface Phonons and Surface Plasmons in Small Crystals. Surf. Sci..

[65] Xiong C., Xiong Y., Zhu H., Zhang Y., Liu Y. (1997). Investigation of Raman Spectrum for Nano-SnO_2_. Sci. China A.

[66] Kumar V., Ayoub I., Sharma V., Swart H.C. (2023). Optical Properties of Metal Oxide Nanostructures.

[67] Chu D., Mo J., Peng Q., Zhang Y., Wei Y., Zhuang Z., Li Y. (2011). Enhanced Photocatalytic Properties of SnO_2_ Nanocrystals with Decreased Size for ppb-Level Acetaldehyde Decomposition. ChemCatChem.

[68] Sato K., Yokoyama Y., Valmalette J.C., Kuruma K., Abe H., Takarada T. (2013). Hydrothermal Growth of Tailored SnO_2_ Nanocrystals. Cryst. Growth Des..

[69] Sangeetha P., Sasirekha V., Ramakrishnan V. (2011). Micro-Raman Investigation of Tin Dioxide Nanostructured Material Based on Annealing Effect. J. Raman Spectrosc..

[70] Tameh M.S., Gladfelter W.L., Goodpaster J.D. (2025). Unraveling Surface Chemistry of SnO_2_ Through Formation of Charged Oxygen Species and Oxygen Vacancies. Int. J. Quantum Chem..

[71] Tauc J. (1968). Optical Properties and Electronic Structure of Amorphous Ge and Si. Mater. Res. Bull..

[72] Anandan K., Rajendran V. (2010). Size Controlled Synthesis of SnO_2_ Nanoparticles: Facile Solvothermal Process. J. Non-Oxide Glas..

[73] Mayandi J., Marikkannan M., Ragavendran V., Jayabal P. (2014). Hydrothermally Synthesized Sb- and Zn-Doped SnO_2_ Nanoparticles. J. Nanosci. Nanotechnol..

[74] Godlaveeti S.K., Somala A.R., Sana S.S., Ouladsmane M., Ghfar A.A., Nagireddy R. (2022). Evaluation of pH Effect of Tin Oxide (SnO_2_) Nanoparticles on Photocatalytic Degradation, Dielectric and Supercapacitor Applications. J. Clust. Sci..

[75] Li D., Sun Y., Pei J., Yu X., Tian Z., Xu H. (2024). Au–SnO_2_ Resonator for SERS Detection of Ciprofloxacin. Microchem. J..

[76] Fernández-Vidal J., Gómez-Marín A.M., Jones L.A.H., Yen C.-H., Veal T.D., Dhanak V.R., Hu C.-C., Hardwick L.J. (2022). Long-Life and pH-Stable SnO_2_-Coated Au Nanoparticles for SHINERS. J. Phys. Chem. C.

[77] Zhou Y., Gu Q., Qiu T., He X., Chen J., Qi R., Huang R., Zheng T., Tian Y. (2021). Ultrasensitive Sensing of Volatile Organic Compounds Using a Cu-Doped SnO_2_–NiO p–n Heterostructure That Shows Significant Raman Enhancement. Angew. Chem. Int. Ed..

[78] Liu B.Y., Zhang W., Lv H.M., Zhang D., Gong X. (2012). Novel Ag-Decorated Biomorphic SnO_2_ Inspired by Natural 3D Nanostructures as SERS Substrates. Mater. Lett..

[79] Jiang L., Yin P., You T., Wang H., Lang X., Guo L., Yang S. (2012). Highly Reproducible Surface-Enhanced Raman Spectra on Semiconductor SnO_2_ Octahedral Nanoparticles. ChemPhysChem.

[80] Hou J.L., Jia F.X., Xue X.X., Chen L., Song W., Xu W.Q., Zhao B. (2012). Surface-Enhanced Raman Scattering of Molecules Adsorbed on SnO_2_ Nanoparticles. Chem. J. Chin. Univ..

[81] Podstawka E., Kudelski A., Proniewicz L.M. (2007). Adsorption Mechanism of Physiologically Active L-Phenylalanine Phosphonodipeptide Analogues: Comparison of Colloidal Silver and Macroscopic Silver Substrates. Surf. Sci..

[82] Podstawka E., Ozaki Y., Proniewicz L.M. (2004). Part I: Surface-Enhanced Raman Spectroscopy Investigation of Amino Acids and Their Homodipeptides Adsorbed on Colloidal Silver. Appl. Spectrosc..

[83] Podstawka E., Borszowska R., Grabowska M., Drąg M., Kafarski P., Proniewicz L.M. (2005). Investigation of Molecular Structure and Adsorption Mechanism of Phosphonodipeptides by Infrared, Raman, and Surface-Enhanced Raman Spectroscopy. Surf. Sci..

[84] Proniewicz E., Tąta A., Starowicz M., Wójcik A., Pacek J., Molenda M. (2021). Is the Electrochemical or the Green Chemistry Method the Optimal Method for the Synthesis of ZnO Nanoparticles for Applications to Biological Material? Characterization and SERS on ZnO. Colloids Surf. A.

[85] Proniewicz E., Vijayan A.M., Surma O., Szkudlarek A., Molenda M. (2024). Plant-Assisted Green Synthesis of MgO Nanoparticles as a Sustainable Material for Bone Regeneration: Spectroscopic Properties. Int. J. Mol. Sci..

[86] Proniewicz E., Tąta A., Szkudlarek A., Świder J., Molenda M., Starowicz M., Ozaki Y. (2019). Electrochemically Synthesized γ-Fe_2_O_3_ Nanoparticles as Peptide Carriers and Sensitive and Reproducible SERS Biosensors: Comparison of Adsorption on γ-Fe_2_O_3_ versus Fe. Appl. Surf. Sci..

[87] Podstawka E., Ozaki Y., Proniewicz L.M. (2005). Part III: Surface-Enhanced Raman Scattering of Amino Acids and Their Homodipeptide Monolayers Deposited onto Colloidal Gold Surface. Appl. Spectrosc..

[88] Tąta A., Szkudlarek A., Pacek J., Molenda M., Proniewicz E. (2019). Peptides of Human Body Fluids as Sensors of Corrosion of Titanium to Titanium Dioxide: SERS Application. Appl. Surf. Sci..

[89] Tąta A., Szkudlarek A., Kim Y., Proniewicz E. (2017). Interaction of bombesin and its fragments with gold nanoparticles analyzed using surface-enhanced Raman spectroscopy. Spectrochim. Acta A.

